# Novel Human Metapneumovirus Sublineage

**DOI:** 10.3201/eid1201.050772

**Published:** 2006-01

**Authors:** Barbara Huck, Gesa Scharf, Dieter Neumann-Haefelin, Wolfram Puppe, Josef Weigl, Valeria Falcone

**Affiliations:** * University Hospital Freiburg, Freiburg, Germany;; † University Hospital Schleswig-Holstein, Kiel, Germany

**Keywords:** human metapneumovirus, HMPV, respiratory infections, phylogeny, dispatch

## Abstract

In a pediatric surveillance network, 287 (5.1%) of 5,580 specimens from patients with acute respiratory infections tested positive for human metapneumovirus (HMPV). Phylogenetic analysis of N- and F-gene sequences of identified HMPV showed that 30% belonged to a novel phylogenetic cluster.

Human metapneumovirus (HMPV), a newly discovered member of the family *Paramyxoviridae*, pneumovirus subfamily, was first isolated in the Netherlands in 2001 (*1*). Since then, a variety of reports have confirmed the worldwide prevalence of HMPV and identified this virus as an important respiratory pathogen in young children (*2**–**5*). Sequence analysis of several isolates has identified 2 major genetic lineages (subtypes A and B) that can be divided into subgroups A1, A2, B1, and B2 (*6**,**7*). Since a growing number of sequence data are available, we initiated a systematic analysis of HMPV genotypes within subtypes A and B to better characterize HMPV variability.

## The Study

From October 2002 to June 2004, nasopharyngeal aspirates (NPAs) from 5,580 pediatric patients <16 years of age (median 22 months) were collected by a pediatric infectious diseases network on acute respiratory infections (PID-ARI.net). This surveillance system comprises 3 study areas in northern (Kiel), midwestern (Mainz), and southern (Freiburg) Germany. Two percent of the total German pediatric population are under surveillance. Those identified by this network included hospitalized children (87.6%) and outpatients (12.4%) with symptoms of acute upper or lower respiratory tract infections. The most common signs and symptoms were cough, rhinorrhea, pharyngitis, otitis media, exanthema, fever, rales, and retractions.

Respiratory pathogens were detected by multiplex reverse transcription–polymerase chain reaction (RT-PCR) and enzyme-linked immunosorbent assay, based on methods described previously (*8*), including primers specific for HMPV, respiratory syncytial virus, parainfluenza viruses 1–3, rhinoviruses, influenza A and B viruses, adenoviruses, and enteroviruses, as well as noncolonizing bacterial pathogens of the respiratory tract. The primers used for HMPV detection correspond to those published previously (*9*). The L-gene primers were used from October 2002 to June 2003; the N-gene primers, adapted versions of the published (NL-N) primers, were used from July 2003 to June 2004. HMPV was detected in 287 NPAs: 51 (1.9%) of 2,599 specimens in the first season (October 2002–June 2003), and 236 (7.9%) of 2,981 specimens in the second season (July 2003–June 2004). RNA was extracted by using the QIAamp Viral RNA Mini-Kit (Qiagen GmbH, Hilden, Germany). Two independent regions of the nucleocapsid (N, nucleotide [nt] 454–878) and fusion protein gene (F, nt 3,624–4,130) were selected for phylogenetic analysis and amplified with the primer pairs: N-f (5´-CCYTCAGCACCAGACACACC-3´), N-r (5´-AGATTCAGGRCCCATTTCTC-3´) and F-f (5´-GTYAGCTTCAGTCAATTCAACAGAAG-3´), F-r (5´-CCTGTGCTGACTTTGCATGGG-3´) by using the Qiagen OneStep RT-PCR Kit (Qiagen GmbH). We used 5 μL RNA in a volume of 50 μL, a primer concentration of 0.6 mmol/L, and the following reaction conditions: 30 min at 50°C, 15 min at 95°C, 35 cycles of 30 s at 94°C, 30 s at 58°C, and 1 min at 72°C, and a final incubation of 10 min at 72°C. Nucleotide sequences from amplified F and N gene products purified with the QIAquick PCR Purification Kit (Qiagen GmbH) were determined by using the BigDye V 3.1 cycle sequencing kit (Applied Biosystems, Foster City, CA, USA) on an automated ABI 3730 XL capillary sequencer (GATC, Konstanz, Germany). Sequences were aligned with prototype HMPV strains from Canada and the Netherlands (GenBank accession numbers AY297748, AY145295, AY145277, AY297749, AY355324, AY145301, AY145276, AY145299, AY145274, AY145294, AY145286, AY355335, AY525843, AF371337, AY530095) and sequences of the avian metapneumovirus C (AY590688), by using the ClustalW algorithm of the Megalign software (Lasergene, DNA Star, Madison, WI, USA). Neighbor-joining trees were generated with neighbor-joining and the Kimura 2 parameter substitution model by using MEGA software (*10*); 1,000 bootstraps were performed on the neighbor-joining trees. Additional phylogenetic testing of the datasets was performed by maximum likelihood (ML) analysis with the DNAml software of PHYLIP (PHYLIP [Phylogeny Inference Package] Version 3.6; distributed by J. Felsenstein, Department of Genome Sciences, University of Washington, Seattle, WA, USA). One hundred bootstrap replicates were calculated on the resulting phylogenetic trees by using the Seqboot and Consense programs of the PHYLIP package. The datasets for the 2 seasons were analyzed separately. Partial N-gene and F-gene sequences of 424 and 506 nt, respectively, were obtained for 230 NPA samples. In 57 samples that initially tested positive for HMPV, the viral genotype could subsequently not be determined because of insufficient NPA, unsuccessful recovery of viral RNA after shipping and extended storage, or differences in the extraction methods and RT-PCR.

**Figure 1:**
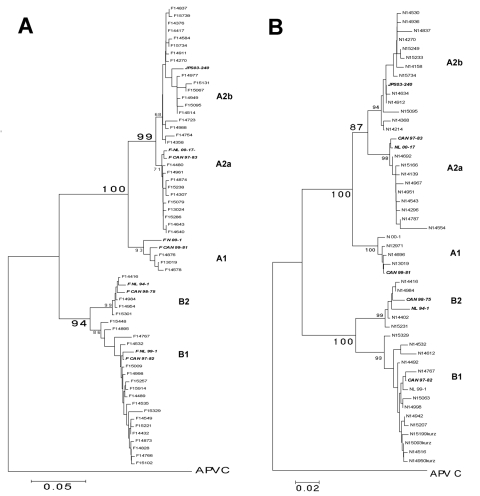
eighbor-joining phylogenetic trees of human metapneumovirus (HMPV). A) Partial F gene (506-nucleotide [nt] fragment). B) Partial N gene (424-nt fragment) of 191 HMPV strains recovered in Germany during the 2003–2004 season. Bootstrap resampling was applied (n = 1,000) with random sequence addition. Bootstrap values based on the consensus tree are plotted at the main internal branches to show support values. Sequences from the avian metapneumovirus C were included in the analysis and used as outgroup. Isolates from the Netherlands, Japan, and Canada were additionally included in the analysis.

Phylogenetic analysis of the N-gene fragment by the neighbor-joining method performed on 191 samples from the season 2003–2004 (Figure 1B) confirmed the existence of 2 main genetic lineages, A and B, and the 2 formerly reported subgroups, B1 and B2, in lineage B. However, lineage A appeared to consist of 3 subclusters (Figure 1). In fact, we found 2 clusters, tentatively named A2a and A2b, within the formerly reported subgroup A2. This partition of subgroup A2 is supported by high bootstrap values (94% and 98%) comparable to those found for the widely accepted partition of A into A1 and A2. When these sequences were compared in an ML analysis (data not shown), they showed the same tree topology, again supported by high bootstrap values (86%). Moreover, neighbor-joining analysis of the F-gene fragment confirmed the observed partition within the A2 subgroup (Figure 1B) sustained by similar bootstrap values. Therefore, the proposed classification is independent of the calculation model and valid for both gene fragments tested. Analysis of the 39 samples from the 2002–2003 season showed the same tree topology by both neighbor-joining and ML analysis (data not shown). Thus, the cluster A2b was consistently prevalent within 2 consecutive seasons.

The analysis of sequence similarity additionally supported the observed tree topology. For the F-gene fragment, nucleotide identity between groups A and B was 83.6%–87.4%, whereas it was 92.1%–94.3% and 94.0%–95.7% between subgroups A1–A2 and B1–B2, respectively. The sequences within the 3 subgroups A1, B1, and B2 shared a nucleotide identity of 97.1%–99.5%, 97.1%–99.8%, and 98.3%–99.5%, respectively. Reflecting the tree topology, subgroup A2 was the most divergent; sequences shared 92.1%–99.8% nucleotide identity in the F gene. Within clusters A2a and A2b, nucleotide identity of 99.3%–99.8% and 97.1%–99.8% was found, thus confirming the existence of 2 genetically distinct clusters, A2a and A2b. Analysis of the sequences obtained for the N gene yielded comparable results (data not shown).

Phylogenetic analysis of previously published sequences of the HMPV F and N genes of strains from various countries showed that the subtype A sequences cluster with few exceptions in subgroups A2a and A1. None of the fully sequenced prototype isolates (NL 00-1, CAN 98-75, CAN 97-83, NL 99-1) is part of the new cluster A2b. Only 4 isolates from Japan, e.g., JPS03-240 shown in Figure 1 (*11*), were assigned to A2b. In other studies, A2b isolates may have been missed because very short fragments were sequenced (*1**,**3**,**12*). In contrast, 70 (30%) of 230 specimens analyzed in this study belonged to cluster A2b.

**Figure 2:**
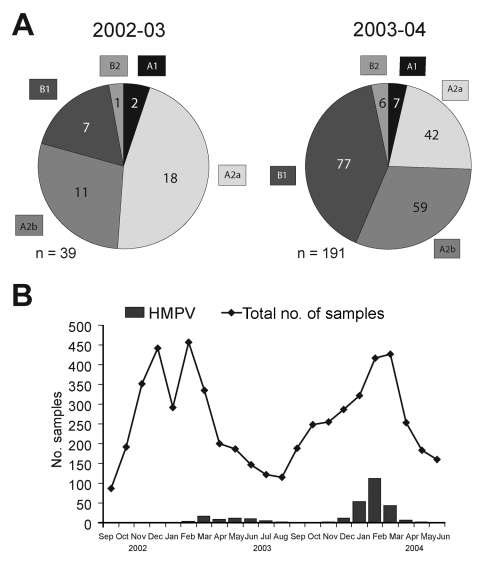
Circulation of human metapneumovirus (HMPV) in Germany in a 2-year period from October 2002 to June 2004. A) Distribution of HMPV genotypes of 39 patients tested during the 2002–2003 season and 191 patients tested during the 2003–2004 season. B) Seasonal distribution of HMPV-infected patients and overall study admissions by month of admission. Further information about other respiratory pathogens cocirculating during the observed season is available from http://www.pid-ari.net

When we compared the prevalence of the different genotypes during the 2 seasons, we found that HMPV genotypes A1 and B2 were marginally present in both seasons and that the major portion consisted of the A2 and B1 genotypes (Figure 2A). Group A strains were more prevalent than group B strains, in particular, during 2002–2003. Differentiation of the hospitalized patients and the outpatients did not show any significant differences in genotype distribution among the 2 groups. However, we cannot rule out the existence of such a difference because of the small percentage of outpatients (12.4%).

Year-round sampling and testing confirmed a seasonal distribution of HMPV-positive cases (*12*). Seasonal peaks were observed from March to July 2003 and from January to March 2004 (Figure 2B). The rates of HMPV and respiratory syncytial virus detection differed significantly between the 2 years and seemed to be inversely correlated (1.9% vs 15.2% in 2002–2003 and 7.9% vs 11.0% in 2003–2004). Differences in distribution and prevalence of HMPV between the 2 seasons must be interpreted with caution for 2 reasons. First, the number of HMPV-positive specimens from the first year is small. Second, the primers used during the first season demonstrated a variable sensitivity for different genetic lineages (*9*). However, our data are consistent with previous observations of high and low incidence of HMPV in alternating years (*13**,**14*). For respiratory syncytial virus, biannual periodicity with alternating occurrence of minor epidemics (slow onset, low peak, long duration) and major epidemics (rapid onset, high peak, short duration) has been described in temperate climates (*15*). To speculate that HMPV epidemics exhibit a similar pattern of periodicity is tempting, although additional studies performed over longer periods are needed to better define the seasonality of HMPV.

## Conclusions

The molecular epidemiology of the HMPV circulating in Germany during 2 consecutive seasons was distinct from the previously proposed classification scheme. By genotyping >200 samples, we confirmed the existence of the 2 main HMPV subtypes A and B and of 4 minor subgroups. However, subgroup A2 was more divergent than reported to date. Phylogenetic analysis of both the F and the N genes showed a further bipartition of subgroup A2. Thus, we identified 2 new genetic clusters, designated A2a and A2b. Comparison of HMPV prevalent in Germany during 2 seasons showed cocirculation of all described genotypes with subtype A predominating over subtype B, thus confirming previous reports (*15*). Within subtype A, subgroup A2 accounted for most cases. Moreover, approximately one third of all genotypes were classified as A2b in both seasons. Given this high prevalence, genotype A2b should be considered in HMPV primer design for diagnostic assays. The finding that sequences from Japanese HMPV isolates belong to genotype A2b (*11*) supports the idea that this novel sublineage is not locally or temporarily restricted but might be prevalent worldwide.
